# The expression of PLK-1 in cervical carcinoma: a possible target for enhancing chemosensitivity

**DOI:** 10.1186/1756-9966-28-130

**Published:** 2009-09-23

**Authors:** Yuan Zhang, Yu Liu, Yuan-Xian Yang, Jia-Hong Xia, Hong-Xiu Zhang, Hua-Bin Li, Chun-Zhao Yu

**Affiliations:** 1 Department of Obstetrics and Gynecology, Union Hospital, Tongji Medical College, Huazhong University of Science and Technology, No. 1277 Jiefang Avenue, Wuhan, Hubei, PR China; 2 Department of Medicine, Feinberg Medical School, Northwestern University, 745 N Fairbanks, Chicago, IL, USA; 3 Department of Surgery, Union Hospital, Tongji Medical College, Huazhong University of Science and Technology, No 1277 Jiefang Avenue, Wuhan, Hubei, PR China; 4 Department of Surgery, The First Affiliated Hospital, Nanjing Medical University, No 300 Guangzhou Road, Nanjing, Jiangsu, PR China

## Abstract

**Background:**

Polo-like kinase-1 (PLK-1) is reported to be upregulated in a variety of human tumors and is implicated in cell proliferation and survival. However, its importance in cervical carcinoma has not yet been fully elucidated.

**Methods:**

We examined PLK-1 expression in cervical carcinoma tissues using immunohistochemical staining. Furthermore, we blocked PLK-1 expression in HeLa cells using specific siRNA and detected the cell cycle, cell proliferation and chemosensitivity using western blotting, MTT and flow cytometry.

**Results:**

We provide evidence that expression of PLK-1 exists in human cervical carcinoma tissues and establish an association with tumor size. Furthermore, we show that PLK-1 knockdown by transfection of siRNA induces accumulation of HeLa cells in the G2/M cell cycle phase and enhances cisplatin-induced apoptosis.

**Conclusion:**

Our results indicate that PLK-1 production in HeLa cells might be critical in determining whether cells survive or undergo apoptosis. Therefore, targeting PLK-1 might be a promising strategy for enhancing sensitivity to chemotherapeutic reagents in cervical carcinoma.

## Background

Cervical carcinoma is a common malignancy worldwide and its incidence has been increasing gradually. It poses a significant health problem, especially in regions such as Asia and North America. Despite advances in diagnostic and treatment modalities, the proportion of failed treatments is still significant, with reported rates of 15.6% to 58% [1]. To date, chemotherapy is the mainstay of treatment modalities for cervical carcinoma and cisplatin has proven to be the most effective single cytotoxic agent for the treatment of advanced or recurrent cervical cancer [2]. However, the response rate is about 23%, due to chemoresistance. Therefore, it is necessary to develop a novel strategy to overcome the chemoresistance of cervical carcinoma and improve clinical efficiency and prognosis.

Although the molecular events responsible for the pathogenesis of cervical carcinoma remain to be elucidated, the final common pathway of carcinogenesis appears to be a disruption of the mechanisms involved in the regulation of cell cycle progression, leading to uncontrolled cell proliferation [3]. Critical cellular signaling underlying the regulation of cell cycle progression has been implicated in a number of cancers. With regard to tumorigenesis, it is worth noting that polo-like kinase 1 (PLK-1), a mitotic cyclin-independent serine-threonine kinase that is believed to be involved in the pathogenesis of numerous carcinomas [4-6], has attracted much attention as a potential therapeutic target.

PLK-1 is a member of the family of polo-like kinases involved in a wide variety of cell cycle processes [7]. In mammalian cells, PLK-1 is primarily localized in the centrosome, where it is responsible for centrosome separation and maturation. PLK-1-specific antibodies introduced into HeLa cells by microinjection prevent centrosome separation and reduce γ-tubulin accumulation, suggesting that PLK-1 functions in regulating centrosome function [8]. PLK-1 is also a target of the G2 DNA damage checkpoint, where it undergoes ubiquitin-dependent proteolysis mediated by the checkpoint protein Chfr, implicating the loss of Plk-1 function as an important response to DNA damage during the G2 phase of the cell cycle [9]. Correspondingly, the elevation of PLK-1 expression occurs in a broad range of human tumors [10,11], and a close correlation has been documented between mammalian PLK-1 expression and progression of endometrial and ovarian cancers [12,13]. Therefore, PLK-1 is implicated as a critical candidate target for understanding the progression of cervical carcinoma and improving chemotherapy. However, little is known about the importance of PLK-1 in the development and management of cervical carcinoma.

To address this issue, we investigated the expression and distribution of PLK-1 in cervical carcinoma tissues. Furthermore, in order to determine the importance of PLK-1 in tumor progression, we investigated the effects of PLK-1 knockdown on the biological characteristics of HeLa cells by taking advantage of small interference RNA (siRNA) against PLK-1. Our results elucidate the pathogenesis of cervical carcinoma and may help to develop a novel strategy to improve the efficiency of chemotherapy delivered to patients with cervical carcinoma.

## Materials and methods

### Immunohistochemical staining

For immunohistochemical staining, thirty-six surgically resected human cervical carcinoma tissue samples were collected from the Department of Obstetrics and Gynecology, Wuhan Union Hospital. The study was approved by the institutional review boards. Immunohistochemical staining was performed according to our previous protocol [14]. Briefly, human tumor tissues were embedded in paraffin and cut into 5-μm sections that were placed onto glass slides. After antigen retrieval, sections were stained for the expression of PLK-1 (BD Biosciences, San Diego, CA) (1:100)detected by streptavidin-biotin-horseradish peroxidase complex formation. Tumor sections stained for IgG instead of primary antibodies were used as the negative control. The immunoactivities of PLK-1 were ranked according to the percentage of positive tumor cells: score 3 (> 75%), score 2 (25-75%), score 1 (< 25%), and score 0 (negative).

### Cell culture, transient transfection, RNA interference, and cisplatin treatment

HeLa cells were cultured in RPMI 1640 supplemented with 10% fetal calf serum (FCS) (Invitrogen, Carlsbad, CA,). Plasmid construction and transfection were performed as previously described [4]. Briefly, PLK-1 cDNA was cloned into the pcDNA3.2-DEST vector (Invitrogen), and the resulting expression plasmid (pcDNA3.2-DEST-Plk1) was verified according to the reference sequence. PLK-1 (GenBank accession no. NM_005030) siRNAs, targeting regions of the Plk-1 transcript at positions 362-384, were also used in this study. HeLa cells were transfected at 70% to 90% confluency using PLK-1 plasmid DNA (up to 4 μg) mixed with Lipofectamine 2000 (Invitrogen) at a DNA (μg)/lipid (μL) ratio of 1:2.5. Similarly, PLK-1 silencing was performed by transfecting HeLa cells with PLK-1 siRNA plasmids. At 4-6 h post-transfection, the plasmid- or siRNA-containing medium was replaced with normal culture medium containing 10% FCS, and the cells were incubated in a 5% CO_2 _incubator at 37°C. Transfected cells were then cultured in fresh medium for up to 12-36 h and harvested for gene expression and other assays. For cisplatin treatment, cisplatin (4 μg/ml) was added to HeLa cells, with DMSO as control. The time point chosen for the addition of cisplatin to the transfected cells was 24 h after transfection, and was based on preliminary experiments (data not shown).

### Quantitative RT-PCR analysis for mRNA levels

Real-time RT-PCR was performed as detailed in our previous report [14]. Briefly, total RNA was extracted with TRIzol reagent (Invitrogen), following the manufacturer's instructions. Reverse transcription (RT) was performed, and the cDNA was synthesized from 2 μg of total RNA by using an oligo (dT)18 primer and M-MLV reverse transcriptase (TAKARA, Syuzou, Shiga, Japan) for quantitative PCR. Expression of mRNA was determined using the ABI PRISM 7300 Detection System (Applied Biosystems, Foster City, CA) and SYBR Premix Taq™ (TAKARA). The sequences of the primers were as follows: PLK1 (NM_005030) forward: 5'-GGA CTA TTC GGA CAA GTA CG-3'; PLK1 reverse: 5'-CGG AAA TAT TTA AGG AGG GTG A-3'; β-actin (NM_001101) forward: 5'-AAG ATG ACC CAG ATC ATG TTT GAG ACC-3'; β-actin reverse: 5'-AGC CAG GTC CAG ACG CAG GAT-3'. The mean value of the replicates for each sample was calculated and expressed as cycle threshold (Ct). The amount of gene expression was then calculated as the difference (ΔCt) between the Ct value of the target gene and the Ct value of β-actin.

### Assessment of cell viability by MTT Assay

Treated or untreated cells were seeded into 96-well plates at 1 × 10^3 ^cells per well overnight and incubated with different concentrations of cisplatin (0 or 4 μg/ml) per treatment. After culture for 24 h, 20 μl MTT dye solution (5 mg/ml) was added to each well and samples were incubated at 37°C for 4 h. The formazan product was dissolved by adding 200 μL of DMSO to each well. The plates were read at 570 nm.

### Immunoblotting analysis

Immunoblotting was performed as previously described [14]. Briefly, treated and untreated HeLa cells were collected and the protein concentrations of lysates were determined by the Bradford method (Pierce, Rockford, IL). Samples containing 10 μg of protein were boiled and subjected to sodium dodecyl sulfate polyacrylamide gel electrophoresis (SDS-PAGE) on 10% Tris-glycine gels and transferred electrophoretically to polyvinylidene fluoride membranes. Primary antibodies (mouse anti-human PLK-1 and β-actin monoclonal antibody, 1:2,000) (Santa Cruz Biotechnology, Santa Cruz, CA) were used, followed by incubation with horseradish peroxidase-linked secondary antibody (goat anti-mouse IgG, 1:1,000). Blots were visualized using an Enhanced Chemiluminescence kit (Cell Signaling, Danvers, MA). Therelative band density of PLK-1 to β-actin was quantified with Bio-Rad Quantity One 1-D Analysis Software (Bio-Rad, Hercules, CA). The experiment was performed in triplicate.

### Cell cycle and apoptosis analysis by flow cytometry

Cell cycle and apoptosis status of HeLa cells after treatment were determined by flow cytometry. In brief, treated cells were harvested and washed once with ice-cold 0.1 M PBS, fixed with 70% ethanol and stained with PI solution (50 μg/ml propidium iodide, 1 mg/ml RNase). Cells were then analyzed for cell cycle status by flow cytometry (FACScan, Becton Dickinson, USA). To quantify apoptosis, cells were stained with annexin-V and PI using a Vybrant Apoptosis Assay Kit (Invitrogen) according to the manufacturer's instructions.

### Hoechst 33258 staining and activity analysis of caspase-3

The morphological alterations associated with apoptosis were observed in transfected HeLa cells by microscopy using the Hoechst 33258 staining approach. At 36 h post-transfection, cells were fixed (methanol/glacial acetic acid at 3:1) for 15 min at 4°C. Hoechst 33258 (Santa Cruz Biotechnology, Santa Cruz, CA) was added to the well at a concentration of 10 μg/ml, and cells were then incubated for 20 min at 37°C. Before observation, cells were washed three times with PBS. Caspase-3 activation was also tested with the Caspase-3 Fluorescent Assay Kit (R&D, Minneapolis, MN). Transfected cells were harvested for the assay 36 h after transfection, according to the manual.

### Statistical analyses

Immunostaining of tissue sections was analyzed with the Chi-square test. Differences between groups in terms of mRNA analysis, cell proliferation, and apoptosis were analyzed using a two-tailed *t*-test or analysis of variance (ANOVA) using SPSS 13.0 software. The significance level was set at *P *< 0.05.

## Results

### Expression of PLK-1 in human cervical carcinoma tissues

**Figure 1:**
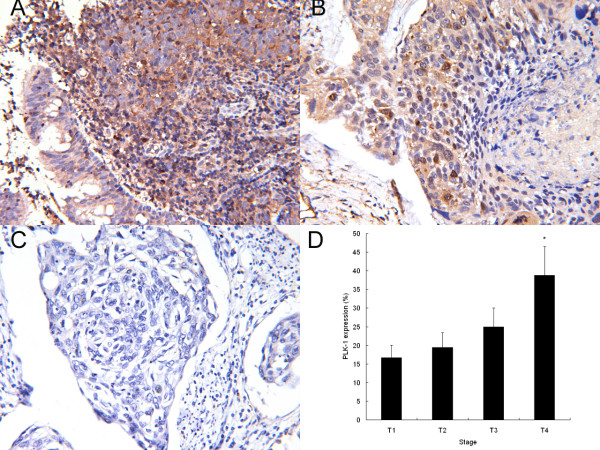
**Immunohistochemical staining of PLK-1 in human cervical carcinoma tissues**. Representative results of immunostaining are presented; cytoplasmic and some nuclear staining can be observed in tumor cells. A, Medium PLK-1 positive staining in human cervical carcinoma tissues (original magnification, 200×); B, low PLK-1 positive staining in human cervical carcinoma tissues (original magnification, 200×); C, PLK-1 negative control staining in human cervical carcinoma tissues (original magnification, 200×); D, Association of PLK-1 expression and primary tumor stage (* *P *< 0.05 compared to other group).

To investigate the presence of aberrant PLK-1 expression in human cervical carcinoma tissues, we examined PLK-1 expression by immunohistochemical staining. The clinical pathologic characteristics of specimens, including tumor size, lymph node status, tumor grade, distant metastasis and biomarker expression are listed in Table 1. Of the 36 tumor sections, 32 showed positive immunostaining for PLK-1, with a positive rate of 88.9%. Examples of immunostained slides are shown in Fig. 1. Cytoplasmic and some brown nuclear staining in tumor cells served as an index of PLK-1 expression.

**Table 1:** Description of the patient population and PLK-1 expression.

**Characteristic**	**Patients (n = 36)**
Age (Median ± SD)	43.2 ± 15.7
	
Histology	
Undifferentiated	21 (58.3%)
Differentiated	15 (41.7%)
Primary tumor stages	
T(1)	6 (16.7%)
T(2)	7 (19.5%)
T(3)	9 (25%)
T(4)	14 (38.8%)
Nodular metastasis	
Yes	6 (16.7%)
No	30 (83.3%)
Distant metastasis	
Yes	3 (8.3%)
No	33 (91.7%)
PLK-1 expression	
High (score 3)	17 (47.2%)
Middle (score 2)	8 (22.2%)
Low (score 1)	7 (19.5%)
Negative(score 0)	4 (11.1%)

To evaluate the possible importance of PLK-1 in tumor progression, we then evaluated the relationship between PLK-1 intensity and tumor size. Using the Spearman rank correlation test, a statistically significant positive correlation between PLK-1 expression and primary tumor stage (*r *= 0.605, *P *= 0.002) but not metastasis was identified. Our results, therefore, provided clues that the expression of PLK-1 is associated with the local expansion of cervical carcinoma.

### Levels of PLK-1 mRNA and protein in HeLa cells after PLK-1 or siRNA transfection

**Figure 2:**
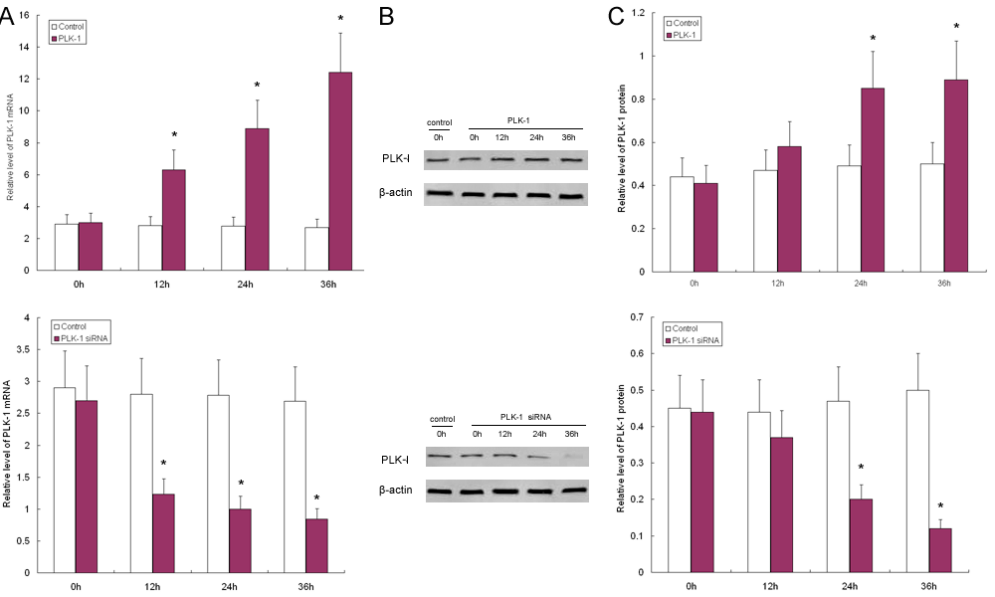
**Alteration of PLK-1 gene and protein expression in HeLa cells after PLK-1 or siRNA transfection**. PLK-1 production in HeLa cells increased after PLK-1 transfection, but was inhibited by siRNA transfection. A, PLK-1 mRNA and protein levels in HeLa cells increased after PLK-1 transfection, but decreased following siRNA transfection. The level of mRNA was determined by real-time RT-PCR. A time-dependent induction was observed; B, Representative results of immunoblotting of PLK-1 expression in HeLa cells were shown. C, PLK-1 protein in HeLa cells increased after PLK-1 transfection, but decreased following siRNA transfection. The level of protein was determined by immunoblotting. A time-dependent modulation was observed. Data were the means of three independent experiments. * *P *< 0.05 compared to the control.

To evaluate the effects of PLK-1 siRNA on the biological characteristics of HeLa cells, we first transfected HeLa cells with the PLK-1 plasmid and PLK-1 siRNA. We harvested cells at different time points (0 h, 12 h, 24 h and 36 h) to measure PLK-1 gene and protein expression. As illustrated in Fig 2, levels of PLK-1 mRNA were significantly elevated after PLK-1 transfection compared to the control cells transfected with empty plasmid, with an increase in expression by 2.2-fold at 12 h, 3.5-fold at 24 h, and 4.7-fold at 36 h (*P *< 0.05). Similarly, an increase was also observed in protein level at 24 h (2.1-fold) and 36 h (2.3-fold). Conversely, siRNA was shown to inhibit PLK-1 mRNA and protein expression. PLK-1 mRNA levels were significantly reduced after PLK-1 siRNA transfection compared to the control cells transfected with empty plasmid, with a decrease of 49% at 12 h, 62% at 24 h, 69% at 36 h (*P *< 0.05). Similar decreases were also observed at the protein level at 24 h (58%) and 48 h (76%). Our results suggest that PLK-1 siRNA transfection into HeLa cells is able to knock-down the expression of PLK-1.

### PLK-1 knock-down by siRNA transfection modulated HeLa cell survival

**Figure 3:**
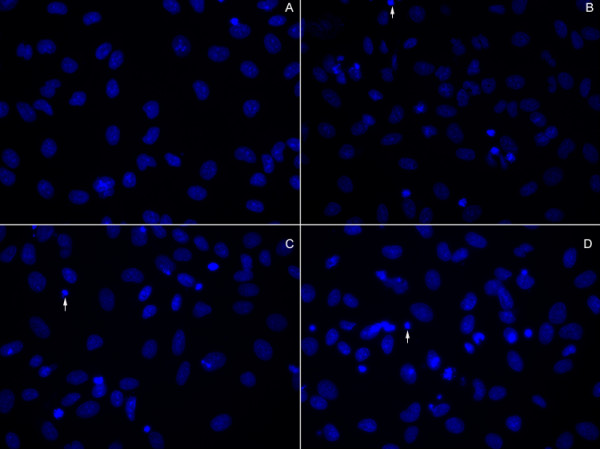
**PLK-1 knock-down by siRNA transfection modulated apoptosis in HeLa cells**. A, Control; B, Cells transfected with PLK-1; C, Cells transfected with PLK-1 siRNA; D, Cells transfected with PLK-1 siRNA and treated with cisplatin (4 μg/ml) (original magnification, 200×); Enhanced apoptosis was demonstrated in B, C and D by typical nuclear condensation after siRNA transfection, as determined by Hoechst staining. Three independent experiments were performed. Representative fluorescent images are presented.

We next evaluated the functional consequences of PLK-1 knock-down on the survival of HeLa cells by morphological examination. As illustrated in Fig 3, we observed enhanced apoptosis in HeLa cells after PLK-1 knock-down with or without cisplatin treatment, as indicated by typical nuclear condensation and cellular shrinkage as determined by Hoechst staining. We then quantitated the number of condensed nuclei per field for several fields. The numbers of condensed nuclei in groups A (control), B (PLK-1), C (PLK-1 siRNA), D (PLK-1 plus cisplatin) were 2.5 (0-7), 6.2 (0-13), 22.7 (5-65), 35.5 (9-77) (condensed nuclei/mm^3^), respectively; the results were significant (*P *< 0.05).

**Figure 4:**
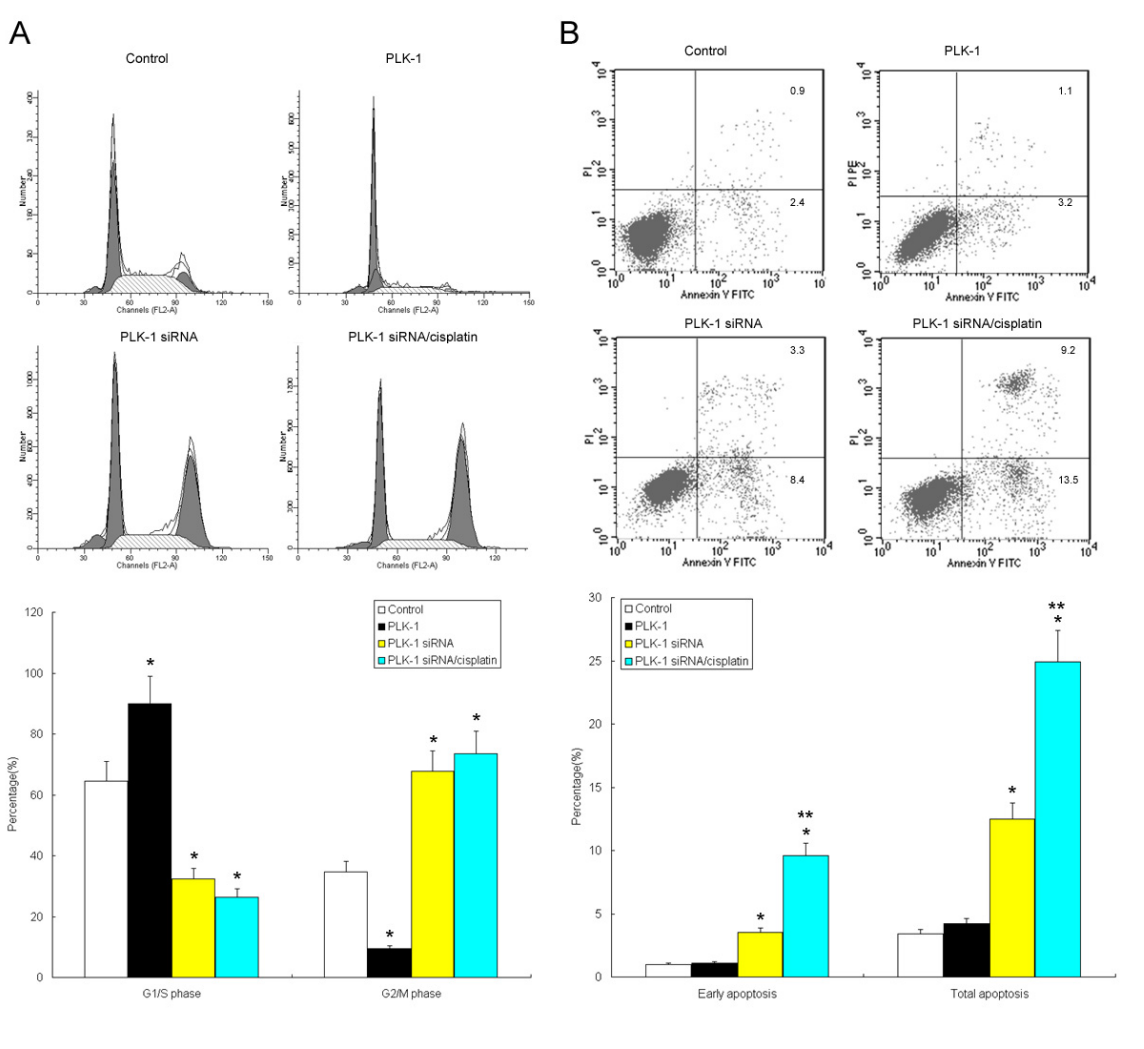
**PLK-1 knock-down modulated cell cycle characteristics and apoptosis in cisplatin-treated HeLa cells**. A synergistic effect with cisplatin treatment (4 μg/ml) was demonstrated. A, PLK-1 siRNA significantly decreased G1/S arrest but enhanced G2/M arrest of HeLa cells; B, PLK-1 siRNA significantly enhanced the apoptosis of HeLa cells, demonstrating a synergistic effect with cisplatin treatment. Representative results of flow cytometric analysis are presented. Data were the means of three independent experiments. * *P *< 0.05, compared to the control cells transfected with empty plasmid; ** *P *< 0.05, compared to the cells transfected with PLK-1 siRNA alone.

To determine whether PLK-1 influences HeLa cell survival, we examined cell cycle characteristics and apoptosis after PLK-1 knockdown by flow cytometry. As shown in Fig. 4, we observed that PLK-1 siRNA significantly decreased G1/S arrest of HeLa cells from 64.5% to 32.5% (*P *< 0.05). Conversely, G2/M arrest of HeLa cells increased significantly from 34.6% to 67.7% (*P *< 0.05). These findings suggested that PLK-1 knockdown contributed to cell cycle progression. In contrast, PLK-1 transfection significantly increased G1/S arrest and decreased G2/M arrest in HeLa cells.

In addition, we also evaluated cell apoptosis after PLK-1 knockdown by double-staining with PI/Annexin-V, followed by flow cytometric analysis. We observed a consistent pro-apoptotic effect of PLK-1 knockdown on HeLa cells. The apoptotic rate of PLK-1 knockdown HeLa cells increased significantly from 4.2% to 12.5% (*P *< 0.05), whereas PLK-1 transfection did not significantly affect HeLa cell apoptosis (Fig. 4). Interestingly, although cisplatin did not drive the cell cycle in combination with PLK-1 siRNA, it acted synergistically with PLK-1 siRNA in inducing cell apoptosis (12.5% *vs. *24.9%, *P *< 0.05).

### PLK-1 knock-down inhibited cell proliferation and increased caspase-3 activity

**Figure 5:**
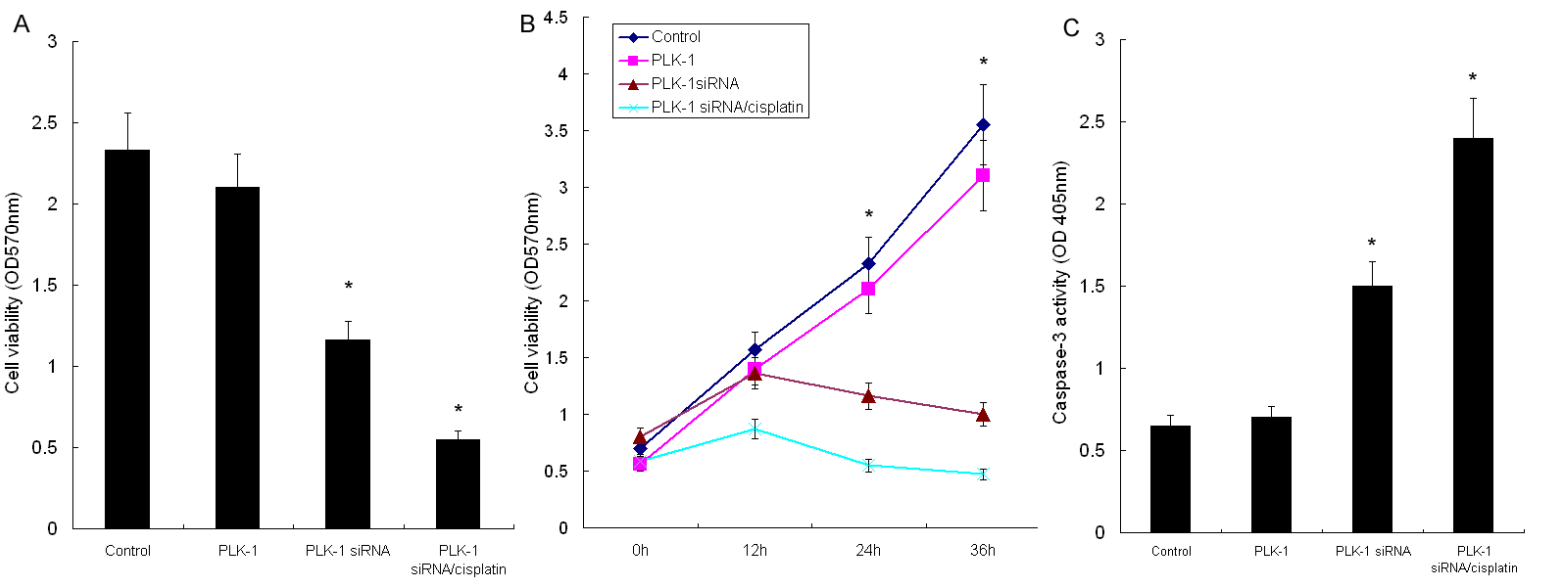
**PLK-1 knockdown by siRNA transfection modulated proliferation and caspase-3 activity in HeLa cells**. A, PLK-1 knockdown significantly inhibited cell proliferation, as determined by MTT assay; B, Cell proliferation curve for four groups of HeLa cells was presented, as determined by MTT assay; C, PLK-1 knockdown significantly increased caspase-3 activity in HeLa cells, as determined by Fluorescent Assay. Data are the means of three independent experiments. * *P *< 0.05 compared to the control cells.

To further determine the effects of PLK-1 siRNA transfection on HeLa cells, we then examined cell proliferation and caspase-3 activity by MTT and fluorescent assay, respectively. As shown in Fig 5, PLK-1 knockdown significantly inhibited cell proliferation, as compared to the control (*P *< 0.05). However, PLK-1 transfection showed no significant effect. After treatment with cisplatin, we observed a synergistic effect of PLK-1 siRNA and cisplatin treatment on HeLa cell proliferation (*P *< 0.05). Furthermore, PLK-1 siRNA significantly increased caspase-3 activity in HeLa cells; caspase-3 activity was further enhanced by cisplatin compared to control and PLK-1 transfected HeLa cells (*P *< 0.05). These results were consistent with those of the morphological examination, flow cytometric analysis and proliferation assays, suggesting that PLK-1 knock-down contributes to the induction of apoptosis in HeLa cells and to enhancing chemosensitivity.

## Discussion

It is well-recognized that PLK-1 plays an important role in cell cycle regulation by functioning in centrosome maturation, spindle formation, mitotic entry, and cytokinesis. When responding to DNA damage, PLK-1 triggers cell cycle arrest in the G2 and M phases, determining cell fate. The significance of PLK-1 has been demonstrated in a variety of tumors. For example, Takai et al. found that expression of PLK-1 in ovarian cancer is associated with histological grade and clinical stage [13]. Feng et al. reported that overexpression of PLK1 is associated with poor survival due to the inhibition of apoptosis via enhancement of survivin levels in esophageal squamous cell carcinoma [15]. PLK-1 has also been demonstrated to be associated with cancer invasion and metastasis [16,17]. However, the significance of PLK-1 in the pathogenesis and management of cervical carcinoma is not well-understood. In the present study, we demonstrated, for the first time, that PLK-1 is expressed in cervical carcinoma with a positive rate of 88.9%, and PLK-1 expression in tumors was associated with primary tumor progression (T stage). Interestingly, we found four samples that were negative for PLK-1 staining, which were later found to be the differentiated samples. These results suggest that PLK-1 expression might be associated with the inactivity of cell mitosis. Therefore, our results indicate that PLK-1 may be a potential target for tumor evaluation and management of cervical carcinoma.

PLK is a well-conserved family that has four known members in humans: PLK1, PLK2, PLK3, and PLK4 [10]. PLK1 expression is regulated during cell cycle progression. Levels are low in G0, G1, and S, but begin to increase in G2 and peak in M phase. PLK-1 has attracted much attention in the field of carcinogenesis and cancer therapy due to its known functions. Blocking PLK-1 through RNA interference has shown promise as a way to intervene in cancer progression [18,19]. RNA interference is a newly discovered cellular pathway for silencing genes in a sequence-specific manner at the mRNA level through the introduction of cognate double-stranded small interfering RNA (siRNA). This method is significantly more efficient than traditional antisense approaches. In our previous study [4], we knocked down PLK-1 production in pancreatic cancer cells by utilizing siRNA transfection, and observed enhanced chemosensitivity to therapeutic agents. To further understand the importance of PLK-1 in the management of cervical carcinoma, we used siRNA transfection to knock down PLK-1 production in HeLa cells.

It has been demonstrated that PLK-1 mRNA expression is elevated in proliferating cells, such as various cancer cell lines and tumors of different origins. Here, we observed the expression of PLK-1 mRNA in HeLa cells. We then transfected PLK-1 plasmids and PLK-1 siRNA into HeLa cells, to evaluate the effects of PLK-1 up- or down-regulation on the biological characteristics of HeLa cells. As we expected, PLK-1 mRNA was significantly elevated after PLK-1 transfection, compared to the control cells transfected with empty plasmid. In contrast, PLK-1 siRNA significantly inhibited PLK-1 production in HeLa cells. These results showed that siRNA transfection of HeLa cells is able to knock down the expression of PLK-1. Based on these findings, we then performed morphological examinations to evaluate the functional consequences of PLK-1 knock-down on HeLa cell survival. We observed enhanced apoptosis in HeLa cells after PLK-1 knock-down with or without cisplatin treatment, as indicated by typical nuclear condensation and cellular shrinkage visualized by Hoechst staining.

Eukaryotic cells have developed a network of checkpoints to ensure their survival and the propagation of accurate copies of the genome to the next generation, even when suffering from a variety of stresses [19]. PLK-1 is a critical component responsible for tumor progression. Silencing PLK1 expression by RNA interference inhibits tumor cell proliferation and induces G2/M arrest. To determine whether PLK-1 influences HeLa survival, we examined cell cycle characteristics and apoptosis after PLK-1 knock-down by using flow cytometry. Importantly, we observed that PLK-1 siRNA significantly decreased the G1/S arrest of HeLa cells from 64.5% to 32.5%. Conversely, G2/M arrest of HeLa cells increased significantly from 34.6% to 67.7%. These findings suggested that PLK-1 contributes to HeLa cell cycle progression.

Currently, cervical carcinoma is the second most common cancer worldwide among women and one of the leading causes of death in relatively young women. Chemotherapy represents a crucial strategy for the management of both primary and recurrent cervical carcinoma [20]. However, some types of cervical carcinoma exhibit limited sensitivity to cytotoxic agents and easily develop drug resistance during long-term chemotherapy [21]. For this reason, enhancing chemosensitivity is essential for improved prognosis. According to the literature, investigating the importance of PLK-1 in the prevention of other cancers, we believe PLK-1 can be considered an important candidate for the enhancement of chemosensitivity in cervical carcinoma.

To examine this possibility, we investigated the apoptosis of HeLa cells after PLK-1 knockdown by RNA interference. Importantly, we observed a consistent pro-apoptotic effect of PLK-1 knock-down in HeLa cells. The apoptotic rate in HeLa cells increased significantly from 4.2% to 12.5% after PLK-1 knockdown, whereas transfection with PLK-1 did not affect HeLa cell apoptosis. Although cisplatin did not drive the cell cycle, when used in combination with PLK-1 siRNA, the compound demonstrated a synergistic effect with PLK-1 siRNA in inducing cell apoptosis (12.5% *vs. *24.9%). Consistently, we observed that PLK-1 knockdown significantly inhibited cell proliferation and induced apoptosis, displaying a synergistic effect with cisplatin treatment. Based on these results, PLK-1 knockdown shows promise as an adjuvant chemotherapy for cervical carcinoma. It will be of great interest to further investigate the possible mechanisms underlying PLK-1-driven cell survival.

In conclusion, we have provided evidence that there is a correlation between overexpressed PLK-1 and the primary cancer stage in cervical carcinoma tissues. To further characterize the role of PLK-1 in the carcinogenesis of cervical carcinoma and the importance of PLK-1 knockdown in the prevention of cervical carcinoma, we investigated the effects of PLK-1 RNA interference on cell cycle characteristics and apoptosis in HeLa cells. We demonstrated that PLK-1 knockdown contributed to G2/M arrest and to the induction of apoptosis in HeLa cells, as well as to the inhibition of cell viability and to the enhancement of chemosensitivity. Therefore, PLK-1 can be thought of as a potential target for preventing cervical carcinoma.

## Conflict of interests

The authors declare that they have no competing interests.

## Authors' contributions

YZ and YL performed the entire experiment. YY and HZ participated in partial experiment (flow cytometric analysis). JX performed the statistic analysis. HL and CY designed the study and prepared the manuscript. All authors read and approved the final manuscript.
